# Endocannabinoid Degradation Enzyme Inhibitors as Potential Antipsychotics: A Medicinal Chemistry Perspective

**DOI:** 10.3390/biomedicines11020469

**Published:** 2023-02-06

**Authors:** Giuseppe Felice Mangiatordi, Maria Maddalena Cavalluzzi, Pietro Delre, Giuseppe Lamanna, Maria Cristina Lumuscio, Michele Saviano, Jean-Pierre Majoral, Serge Mignani, Andrea Duranti, Giovanni Lentini

**Affiliations:** 1 Institute of Crystallography, National Research Council of Italy, Via G. Amendola 122/O, 70126 Bari, Italy; 2 Department of Pharmacy—Pharmaceutical Sciences, University of Bari Aldo Moro, Via E. Orabona 4, 70125 Bari, Italy; 3 Department of Chemistry, University of Bari Aldo Moro, Via E. Orabona 4, 70125 Bari, Italy; 4 Institute of Crystallography, National Research Council of Italy, Via Vivaldi 43, 81100 Caserta, Italy; 5 Laboratoire de Chimie de Coordination du CNRS, 205 Route de Narbonne, CEDEX 4, 31077 Toulouse, France; 6 Université Toulouse, 118 Route de Narbonne, CEDEX 4, 31077 Toulouse, France; 7 CERMN (Centre d’Etudes et de Recherche sur le Médicament de Normandie), Université de Caen, 14032 Caen, France; 8 CQM—Centro de Química da Madeira, MMRG (Molecular Materials Research Group), Campus da Penteada, Universidade da Madeira, 9020-105 Funchal, Portugal; 9 Department of Biomolecular Sciences, University of Urbino Carlo Bo, Piazza del Rinascimento 6, 61029 Urbino, Italy

**Keywords:** endocannabinoid system, FAAH inhibitors, MGL inhibitors, repositioning, drug-likeness, hERG, ligand efficiency metrics

## Abstract

The endocannabinoid system (ECS) plays a very important role in numerous physiological and pharmacological processes, such as those related to the central nervous system (CNS), including learning, memory, emotional processing, as well pain control, inflammatory and immune response, and as a biomarker in certain psychiatric disorders. Unfortunately, the half-life of the natural ligands responsible for these effects is very short. This perspective describes the potential role of the inhibitors of the enzymes fatty acid amide hydrolase (FAAH) and monoacylglycerol lipase (MGL), which are mainly responsible for the degradation of endogenous ligands in psychic disorders and related pathologies. The examination was carried out considering both the impact that the classical exogenous ligands such as Δ^9^-tetrahydrocannabinol (THC) and (−)-trans-cannabidiol (CBD) have on the ECS and through an analysis focused on the possibility of predicting the potential toxicity of the inhibitors before they are subjected to clinical studies. In particular, cardiotoxicity (hERG liability), probably the worst early adverse reaction studied during clinical studies focused on acute toxicity, was predicted, and some of the most used and robust metrics available were considered to select which of the analyzed compounds could be repositioned as possible oral antipsychotics.

## 1. Introduction

Clinical and social psychologists seldom use an ancient Hindu parable to illustrate the seductive potential of our perspective on a complex problem—the well-known story of the blind men and the elephant [Briefly, six wise men were blind and wanted to learn about an animal that they had never met before—an elephant. They went through an organoleptic analysis. Depending on the part of the animal body they touched, each of them produced a different definition (e.g., ‘huge, wrinkled fan,’ said the experimenter who had touched the elephant ears; ‘a big rope,’ said the wise man who had touched the elephant swinging tail; and so on) and a vehement disputation originated since each wise man suspected the others as dishonest persons. All six were telling the truth, but each of them had touched only one part of the animal and therefore knew only that part of the truth] [1]. When a complex problem is concerned, we generally know only a part of the truth while the whole story remains mostly unknown. Mental illness is a complex problem since the human brain is a holistic apparatus: our attempt to explain mental disorders at the synaptic level makes us resemble the wise men who wanted to infer the elephant by analyzing only a part of its body. Anyway, when looking for new drugs to treat mental illness, the best rational way still starts from an alleged biochemical defect occurring at one or several types of synaptic gaps.

The dopamine hypothesis has been the leading theory of most psychotic disorders, and the currently used typical and atypical antipsychotics share a common mechanism of action in antagonism of the dopamine D_2_ receptor [2]. The remarkable case of clozapine, the only atypical antipsychotic effective in psychoses that are otherwise treatment-resistant [3], indicates that multipotent antipsychotics acting at many different receptor sites in the brain may represent a more efficacious treatment than existing drugs [4]. Numerous lines of evidence have highlighted the possible involvement of diverse neurotransmitter pathways, including glutamate, serotonin, γ-aminobutyric acid, adenosine, and acetylcholine [5]. Frequently polypharmacy is prescribed to face the most severe psychotic syndromes despite controversial results about the efficacy of the approach [6,7] and related safety concern [8,9], obviously higher in older patients [10].

**Figure 1:**
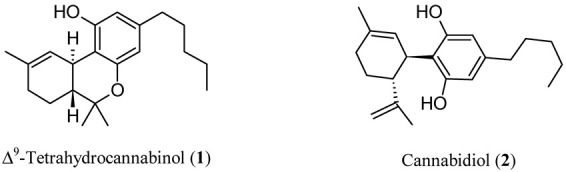
Chemical structures of Δ^9^-tetrahydrocannabinol (THC, **1**) and cannabidiol (CBD, **2**).

In 1997, a “cannabinoid hypothesis of schizophrenia” was also suggested [11], and the increasing body of evidence supporting this new paradigm has been brilliantly reviewed [12]. The endocannabinoid system (ECS) is one of the most relevant neurotransmitter systems in the brain and plays a pivotal role in the regulation of cognitive abilities, mood, stress, and sleep [13]. Relatively fewer explored targets for antipsychotic treatment could be found in ECS. Thinking about cannabinoids, our minds run to the well-known pro-psychotic properties of ∆^9^-tetrahydrocannabinol (THC, Figure 1), the main psychoactive ingredient of cannabis, which acts as an agonist on cannabinoid (CB) receptors (CBR). In addition to its pro-psychotic potential, THC causes an undesirable behavioral tetrad, that is, analgesia, catalepsy, hypothermia, and hypolocomotion. THC synthetic analogs, both agonists and antagonists [14,15], or recreational drugs—the so-called NPS (new psychoactive substances) [16,17]—are generally tainted with severe side effects. The worst is that the activation of CBR of type 1 (CB_1_R) in the central nervous system (CNS) by xenobiotics can lead to irreversible effects [18]. On the other hand, (−)-trans-cannabidiol (CBD, Figure 1), one of cannabis’ main secondary metabolites, seems to be endowed with antipsychotic properties useful to protect against the pro-psychotic effects of THC: depending on its composition, cannabis would act either as Mister Hyde (i.e., a risk factor for psychosis) or as Doctor Jekyll (i.e., an antipsychotic). The hypothesis has been formulated that CBD could be an antipsychotic, with benefits in preventing psychotic disorders, whatever the cause (endogenous or THC-induced) [19].

**Figure 2:**
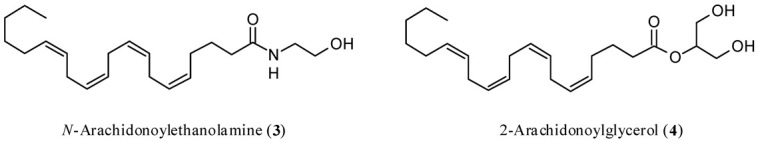
Chemical structures of *N*-arachidonoylethanolamine (anandamide, AEA, **3**) and 2-arachidonoylglycerol (2-AG, **4**).

In a randomized, double-blind controlled clinical trial, CBD exerted antipsychotic properties comparable to the reference drug amisulpride [20,21]. Interestingly, the reduction of psychotic symptoms was significantly associated with an increase in the serum concentrations of *N*-arachidonoylethanolamine (anandamide, AEA, Figure 2), which is the most important endogenous ligand of CBR, and this outcome was found only in patients treated with CBD. The results indicated that, at least in part, the antipsychotic activity of CBD was due to the inhibition of the enzymes physiologically devoted to the degradation of AEA [22], thus acting as an indirect agonist. This finding agrees with the observation that both increased availability of CB_1_R and upregulation of AEA seem beneficial, although the underlying mechanisms are mostly elusive. The evidence supporting the possible protective role of AEA in schizophrenia has been reviewed [23].

The inhibition of enzymes responsible for the degradation of the ECS endogenous ligands might overcome the above obstacles to the systemic use of exogenous substances acting on ECS. EC degradation inhibitors would provide focused action, where and when necessary, by acting as endogenous CBRs ligand modulators. Since the available clinical data on EC potentiators as antipsychotics are relatively scarce and essentially limited to CBD, we conceived this perspective as an attempt to envision which of the EC degradation most considered inhibitors so far might be repositioned as possible oral antipsychotics based on their predicted drug-likeness and safety. After a dutiful short survey on the ECS architecture and functions, we shall review the most studied, clinically relevant EC metabolizing enzyme inhibitors reporting their corresponding main pharmacological activities. Then, we shall predict ADMET properties and calculate structure descriptors generally related to drug-likeness and probability of success as CNS acting agents for each of them using some of the most robust medicinal chemistry predicting tools today available.

The purpose of this narrative review is to select some of the analyzed compounds as either candidates for repositioning as orally active antipsychotics or starting compounds for structural simplification/optimization studies to reduce toxicity and improve selectivity.

The information presented in this perspective was acquired through consultation of the PubMed^®^, Reaxys^®^, and SciFinder^®^ Scholar databases.

## 2. Endocannabinoid System

The exhaustive description of ECS architecture and functioning is beyond the scope of this work; more details can be found elsewhere [24,25]. The right functioning of the ECS is related to the natural balance established between its main components, which are CBRs, the endogenous ligands binding them, and the enzymes involved in the synthesis, transport, and degradation of ECs. A disruption of the physiological activity of this system (i.e., modifications in the expression of receptors or the functions of enzymes) is associated with various pathologies. This situation, therefore, is the basis for therapeutic pharmacological opportunities founded on drugs able to interact naturally with ECS [26,27,28,29,30].

The discovery of CBRs and the main endogenous ligands is relatively recent, as the first one, CB1R, was identified in the second half of the 1980s [31], while the second receptor, namely CB_2_R, was discovered a few years later [32]. The two targets differ in their corresponding main functions, signaling processes, and structural aspects [33,34]. Their signal neurobiology and tissue distribution are also different, being the CB_1_R mainly expressed in the CNS (mostly in the basal ganglia, cerebellum, cortex, and hippocampus), whereas CB_2_R is particularly present in the immune system (mostly in B-cells and natural killers) [35]. Overall, it is demonstrated that CBR, through their activation, performs a key role in inducing activation or depression of neurotransmission by the inhibition of adenylate cyclase, which determines a decrease in cyclic adenosine monophosphate levels, or, only in the case of CB_1_R, by the coupling with ion channels [35,36]. A careful analysis of the above characteristics, in particular those related to the different tissue distribution, is important when envisioning a pharmacological therapy aiming at a selectivity of action and the consequent reduction of undesired effects.

The main and most studied CBR endogenous ligands are AEA [37] and 2-arachidonoylglycerol (2-AG) [38,39] (Figure 2).

Both ligands are produced on demand from membrane phospholipids to satisfy contingent physiological needs due to intense neuronal activation [40,41]. AEA and 2-AG act through a retrograde or non-retrograde signaling pathway. Their half-life is short (a few minutes) as a rapid carrier-mediated diffusion occurs in the cells where they are metabolized [40,41]. It is very interesting to consider that ECs-mediated retrograde signaling is involved in the excitatory or inhibitory processes related to the modulation of neurotransmitters, such as glutamate or γ-aminobutyric acid [41,42,43,44], through short-term and long-term neuroplasticity (taking some seconds and some minutes, respectively) physiological processes [45,46]. The first is involved in processes such as depolarization-induced suppression of inhibition and depolarization-induced suppression of excitation through the inhibition of voltage-gated Ca^2+^ channels, whereas the second one leads to the long-term depression phenomenon through a CB_1_R repeated stimulation of these brain circuits. Consequently, CBR has to be considered a potential drug target for the prevention and treatment of neurologic pathologies, in particular, in the case of CNS involvement [47].

AEA is biosynthesized by the *N*-acyl phosphatidylethanol-selective phospholipase D [48]. It acts as a total or partial agonist of the CB_1_R and has low activity toward CB_2_R [49]. AEA comes up against rapid degradation due to its capture by a transporter [50,51], as occurs in the extracellular space of brain neurons and astrocytes [29], which is followed by the degradative action mainly carried out by fatty acid amide hydrolase (FAAH) [52,53,54,55], a homodimer integral membrane protein. The functional component of the enzyme consists of a catalytic triad formed by the amino acids Lys142-Ser217-Ser241, with the latter determining the nucleophilic attack on the electrophilic carbonyl group of AEA through the hydroxy group [56].

The biosynthesis of 2-AG begins with diacylglycerols and hydrolysis operated by the diacylglycerol lipase isoform α or β [57,58]. It acts as a full agonist of both CB_1_R and CB_2_R [59]. In addition, in the case of 2-AG, therefore, the molecule is captured by a transporter with characteristics identical or similar to those shown by AEA [60], which causes internalization and subsequent hydrolysis mainly by monoacylglycerol lipase (MGL) [61,62,63], an enzyme belonging to the α/β hydrolase superfamily. The mechanism involves the participation of various amino acids, which contribute to the initial preparatory phase aimed at catalytic activity by the Ser122-Asp239-His269 triad, where the serine residue is responsible for the nucleophilic action towards the carbonyl group of the substrate [64,65].

Taken together, the ECS certainly constitutes a reference model for drug discovery endeavors aimed at finding ideal molecules without the undesirable effects caused by the direct activation of CBRs [66]. With this goal, a fundamental role is played by compounds able to inhibit the enzymes that degrade natural ligands.

## 3. FAAH and MGL Inhibitors

Of particular interest is the discovery of the first FAAH enzyme inhibitors dates back just over twenty years ago, while those of MGL appeared a few years later. Since then, there have been numerous studies that have made it possible to expand the panorama of molecules available to the scientific community. The compounds that have emerged over time have the common need to be excellent substrates for enzymes. Therefore, all contain a reactive group able to favor the nucleophilic reaction. However, they differ from each other not only for the type of inhibition expressed (reversible vs. irreversible) but, above all, for the structural characteristics, which allow the opportunity to have various classes, and for potential therapeutic applications. A distinct description of the classes of FAAH and MGL inhibitors will be made here, in particular on those owing a therapeutic potential related to pathologies that may have a direct or indirect influence on psyche disorders. For anything not covered here, please refer to some excellent previously reported reviews [29,66,67,68,69,70].

**Figure 3:**
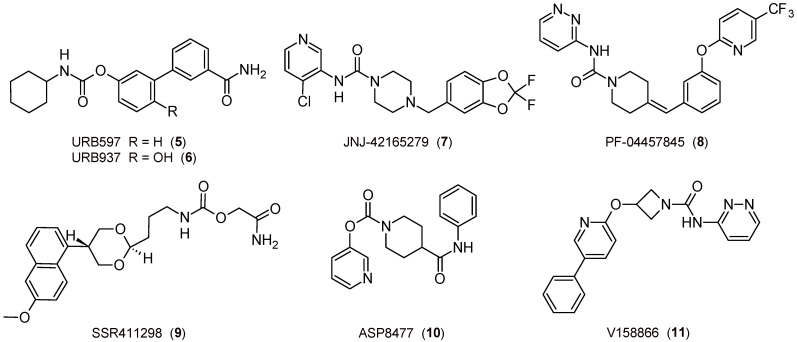
The 2D structures of selected FAAH inhibitors.

The first study on the topic was published about twenty years ago, when covalent and irreversible carbamate FAAH inhibitors, such as the pharmacological tool URB597 (Figure 3), showed relevant anxiolytic-like and antidepressant-like properties related to the indirect activation of CB_1_R [71]. This discovery then opened the way to numerous further related opportunities, such as the possibility of using molecules of the URB series for new experimental models for depression [72,73], but also to test compounds belonging to different classes. The covalent and slow reversible piperazine-urea-based inhibitor JNJ-42165279 (Figure 3) [74] has been characterized and tested in clinical trials for the treatment of social anxiety disorders [75,76] and major depression. On the other hand, the covalent and irreversible piperidine-urea-based inhibitor PF-04457845 (Figure 3) [77] was able to attenuate the anxiety-inducing effect and entered clinical trials [78]. Finally, the reversible carbamate SSR411298 (Figure 3) exerts anxiolytic-like and antidepressant-like effects [79], but it fails clinical studies on major depression disorders [65].

FAAH inhibitors could be considered to face nicotine or cannabis withdrawal syndromes. With regard to tobacco and cannabis disorders, URB597 has proved useful in reducing nicotine reward, preventing the reinstatement of nicotine use [80], and regulating acute and protracted nicotine withdrawal [81]. PF-04457845 was tested in the treatment of cannabis withdrawal and dependence in men [82].

Potential applications for neuropathic and inflammatory pain have been demonstrated with URB597 [83], which had also passed Phase I clinical trials several years ago (data not available), and URB937 (Figure 3) [84,85,86], which showed peripheral FAAH inhibition without the involvement of CNS. On the other hand, PF-04457845 produced potent CBR-dependent antinociceptive effects in both inflammatory and non-inflammatory arthritic pain models [87], but in related phase II clinical trials, it was not effective [88]. Moreover, JNJ-42165279 showed analgesic properties in a neuropathic pain model [74]. The piperidine-carbamate ASP8477 [89] and the azetidine-urea V158866 derivatives (Figure 3) [90] demonstrated significant analgesic effects in animal models of pain, but even in this case, the phase II clinical trials did not give the expected results [65]. For an overview of the influence of ECS in pain regulation, see [91], while for a FAAH inhibitor clinical perspective, see [92].

**Figure 4:**
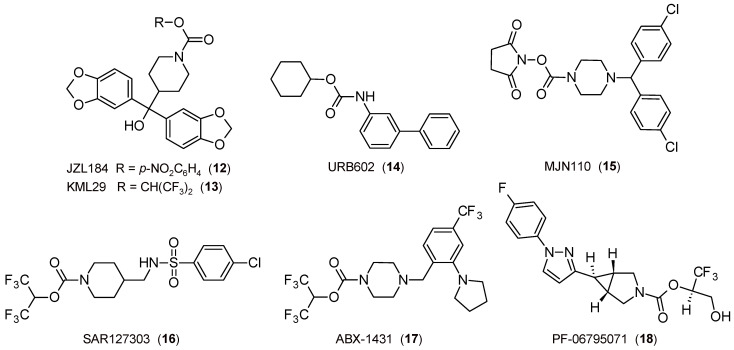
The 2D structures of selected FAAH inhibitors.

The study of MGL inhibitors has also led to the discovery of molecules with interesting profiles. Firstly, concerning anxiety and related disorders, it was observed that the covalent and irreversible piperidine-carbamate JZL184 (Figure 4) [93] attenuates anxiety-like behavior [94,95,96] and exerts an antidepressant-like effect [97].

The first results on the potential of 2-AG in the field of pain were obtained in CB_1_R-dependent stress-induced analgesia employing the covalent and partial reversible carbamate inhibitor URB602 (Figure 4) [42], a molecule with the functional portion reversed in comparison to that present in FAAH inhibitors of the URB series. In the following years, other pharmacological tools have contributed to confirming the importance of inhibition of the MGL enzyme as a therapeutically relevant strategy. It was demonstrated that JZL184 induces a CB_1_R-mediated antinociceptive effect [93,98], whereas the covalent and irreversible piperazine-carbamate MJN110 (Figure 4) [99] alleviates mechanical allodynia [100] and exerts an antihyperalgesic effect through the increase in 2-AG levels [101], as well as the covalent and irreversible piperidine-carbamate KML29 (Figure 4) [102], which produces an antinociceptive activity in pain models [103]. Lastly, experiments carried out with covalent and irreversible pyridazine-carbamate SAR127303 (Figure 4) confirmed not only the ability to produce an analgesic effect in inflammation and pain models but also attenuate the symptoms of epilepsy [104]. The covalent and irreversible piperazine-carbamate ABX-1431 (Figure 4) [105] is effective in pain and neurological diseases and other disorders, such as Tourette syndrome [106], for which phase I clinical studies have been efficacious [65].

Other important results obtained by MGL inhibitors are related to the role of ECS in neuroprotection, which was demonstrated by using URB602 [107] and confirmed some years later through other drugs (for example, see [108]). A similar action is exerted by the covalent and irreversible inhibitor pyrrolidine-carbamate PF-06795071 (Figure 4) in reducing neuroinflammation markers in animal models [109].

**Figure 5:**
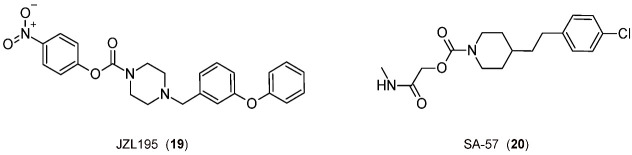
The 2D structures of the selected FAAH/MGL inhibitors.

Finally, it is interesting to consider some exemplary compounds reported as inhibitors of both enzymes. Indeed, covalent piperazine-carbamate-based inhibitors JZL195 [110] and SA-57 [111] (Figure 5) reduce inflammation-induced allodynia at a dose that does not cause side effects [112,113].

## 4. Primum Non Nocere

**Figure 6:**
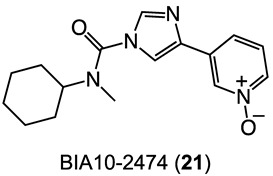
The 2D structure of BIA10-2474 (**21**).

Some of the compounds reviewed in the previous sections entered clinical trials and were generally well tolerated being; the only adverse events the lack of efficacy or the appearance of fewer adverse reactions [66,67]. Thus, they might be expected to be relatively safe as antipsychotics. However, FAAH and MGL inhibitors should be carefully designed and go through accurate biochemical profiling and preclinical investigation before entering further clinical trials. The tragic outcome in the phase I clinical trial on the alleged FAAH inhibitor BIA10-2474 (Figure 6) (one dead volunteer and four others showing severe adverse reactions) does reinforce the caveat [68], but the relative negative outcomes may be puzzling [114]; however, in this specific scenario the misconduct of the trial must certainly be considered [115]. In any case, even where the inhibitors are still in the pipeline, we cannot let our guard down if those inhibitors are candidates for repositioning towards new clinical aims. Some of the FAAH and MGL inhibitors are under clinical evaluation for peripheral clinical activities targeting the peripheral tissues and the spinal cord; higher active doses might be expected for purported inhibitors acting on the brain. In view of the above, human toxicity may be unpredictable from a non-clinical toxicological perspective [116].

Despite what was reported above, medicinal chemists must, to the best of their ability, predict possible toxic liabilities. Several chemoinformatic tools are available to run a preliminary analysis for drug toxicity prediction and usually refer to numerous safety-related properties [117]. To have an overview of the safety of the clinically relevant compounds chosen as candidates for repositioning, we started our analysis with cardiotoxicity (hERG liability), probably the worst early adverse reaction studied during clinical trials focused on acute toxicity [118,119]. To this aim, we employed a recently published ligand-based classifier based on the application of different machine learning algorithms and proved to outperform many predictors commonly used in the literature [120]. More specifically, the model has been developed by using an IC_50_ value equal to 10 μM as the threshold for discerning blockers from non-blockers and applies a consensus strategy based on two different algorithms, namely SVM (Support Vector Machine) and BRF (Balanced Random Forest), to provide the requested predictions. Importantly, all the compounds under investigation fall within the applicability domain of the model [121], hence supporting the reliability of the performed calculations. Table 1 shows the obtained results. Interestingly, none of the considered FAAH inhibitors was predicted as hERG blockers. Noteworthy, these data are in full agreement with the experimental literature available for URB597 [122], JNJ42165279 [74], PF-04457845 [109], and V158866 [90]. As far as the MGL and FAAH/MGL inhibitors are concerned, only three of them were predicted as able to induce hERG-related cardiotoxicity, namely JZL184, ABX-1431, and JZL195. Importantly, these data are again in agreement with the available literature as Grice et al. recently showed that ABX1431 is responsible for a significant hERG activity (IC_50_ = 7 μM) in vitro [105] while McAllister et al. provided experimental evidence that PF-06795071 is not a hERG blocker [109]. To the best of our knowledge, no data are instead available for JZL184 and JZL195. In summary, this preliminary investigation focused on the prediction of hERG-mediated cardiotoxicity indicates that it would be wise not to consider it an ideal candidate for repurposing JZL184, ABX-1431, and JZL195.

## 5. Molecular ‘Beauty’ and ‘Ugly’ Compounds

Medicinal chemists look at their designed and synthesized compounds just like loving moms staring at their kids and generally do not resist the temptation to anthropomorphize their products. When speaking about the drug structural features that may be related to a higher probability of success, they refer to chemical ‘beauty’ (generally linked to a relatively high number of sp^3^ hybridized carbon atoms, Csp^3^), while ‘ugly’ molecules are tainted with some structural defect mostly related to ‘obesity’ (that is, high lipophilicity) that generally reduces that chance. Anthropomorphized jargon in drug design and discovery, while probably oversimplifying concepts, may be useful to make them easily appreciated, reinforce issues, and surrogate long periphrases. At worst, anthropomorphizing drug design is only a bit cryptic for non-aficionados. This is why the above esthetic evaluations have been somewhat codified in dozens of alternative or complementary metrics [123] that, through back-of-the-envelope-calculations allow both naïf and expert scholars to have a rapid outlook of favorable predicted pharmacokinetic (i.e., oral bioavailability) and pharmacodynamic (i.e., efficiency) properties of the designed compound [124,125].

As a first step, we computed the Quantitative Estimate of Drug-likeness (QED), which is an integrative score widely used to estimate the drug-likeness of a given small molecule [126]. In particular, good drug candidates are expected to return a QED > 0.6. The obtained data are reported in Table 2. As expected, most of the known inhibitors exceed this threshold. Few exceptions are represented by one FAAH inhibitor (PF-04457845), three MGL inhibitors (JZL184, KML29, and ABX-1431), and one dual FAAH/MGL inhibitor (JZL195). Noteworthy, all the compounds already predicted as hERG blockers returned low (< 0.6) QED values, thus supporting the idea whereby these inhibitors are not the best candidates as antipsychotics.

Obviously, to have an antipsychotic activity, a given drug must be able to act in the CNS. Furthermore, oral administration is highly desirable. Keeping this in mind, we also computed several descriptors related to the CNS activity as well as oral adsorption in humans using QikProp [125] as a software program, namely: (i) *CNS*, able to provide an estimation of the CNS activity on a −2 (inactive) to +2 (active) scale; (ii) *QPPCaco*, providing an estimation of the Caco-2 cell permeability in nm/sec (i.e., ability to cross the gut-blood barrier; <25 poor, >500 great); (iii) *QPlogBB*, providing an estimation of the brain/blood partition coefficient; (iv) *HumanOralAbsorption*, providing a score indicating a low (1), medium (2) and high (3) qualitative human oral absorption; (v) *PercentHumanOralAbsorption*, providing a score based on multiple linear regression on 0 to 100% scale (>80% is high; <25% is poor) and (vi) *RuleOfThree*; which is the number of violations of the so-called Jorgensen’s rule of three [128]. In particular, compounds responsible for no violations are expected to be orally available. The picture that emerged from data reported in Table 2 puts forward JNJ-42165279 (**7**) as the best candidate for oral antipsychotics. Remarkably, this FAAH inhibitor is predicted to have: (i) a high CNS activity (CNS = 2); (ii) good Caco-2 cell (QPPCaco = 373.210) and blood-brain barrier (QPlogBB = 0.496) permeabilities and (iii) very high oral adsorption in humans (HumanOralAdsorption = 3, PercentHumanOralAbsorption = 87.382 and RuleOfThree = 0). Noteworthy, JNJ42165279 is also responsible for a QED > 0.6 (Table 2) and has been predicted as not able to induce cardiotoxicity (Table 1). Although proven to block hERG [128], worth mentioning is herein also ABX1431 (**17**), which is the only compound [together with JNJ-42165279 (**7**)] predicted as responsible for a high CNS activity (CNS = 2). Finally, interesting data were also provided by MJN110 (**15**), predicted to be responsible for significant CNS activity (CNS = 1) and high oral availability (Table 2).

Finally, we predicted whether the compounds object of the present investigation reacts with one (or more) cytochrome P450 isoforms (among the most important ones, namely CYP1A2, CYP2A6, CYP2B6, CYP2C8, CYP2C9, CYP2C19, CYP2D6, CYP2E1, and CYP3A4). The prediction was performed using as software program CypReact, recently published by Tian et al. [129] and available in the recently published web platform for de novo design DeLA-Drug [130]. The obtained results are shown in Table 3.

All FAAH and MGL selective inhibitors should be substrates of several cytochrome P450 isoforms and thus possibly act as metabolic auto-/heteroinducers. Possible adverse events due to drug-drug metabolic interactions should be considered. Interestingly, both FAAH/MGL inhibitors **19** and **20** were predicted as poor substrates of all cytochrome P450 isoforms, thus performing as the best candidate for repositioning when metabolic liability is concerned.

A complementary analysis may be obtained using three of the most used and robust metrics to classify the selected inhibitors.

(1) As an indicator of promiscuity liability (i.e., the propensity to target not only enzymes but also receptors), the property forecast index has been proposed [131]. This index may be easily obtained as the sum of clogP and number of aromatic rings for each stated compound; as a rule of thumb, property forecast index should be kept below five to reduce possible promiscuity.

(2) CNS penetration may be forecasted from the CNS multiparameter optimization descriptor obtained by a linear combination of six parameters, each weighted one if in the desirable range, 0 when in the undesirable range, and scaled when falling between the targeted values (CNS multiparameter optimization desirability ≥ score, using a scale of 0–6) [132].

(3) As a ligand efficiency evaluator, the lipophilic efficient index is generally considered the most robust descriptor and represents a measure that correlates potency and lipophilicity. It is obtained from the difference between a measure of the potency of the ligand and its clogP and, therefore, can be considered as a measure of the gain in potency net of the (non-specific) entropic contribution due to the mere increase in lipophilicity [133]. An efficient inhibitor should locate its lipophilic efficient index between 5 and 7.

**Figure 7:**
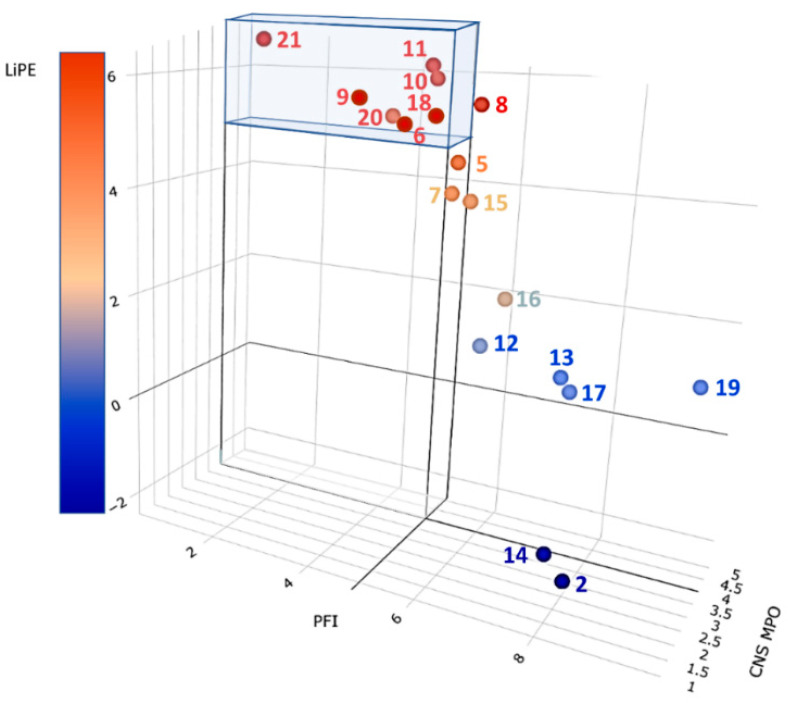
Ligand efficiency metric analysis on the selected inhibitors **2**, **5–21**. Compounds falling within the colored volume are expected to display the highest efficiency, selectivity, and brain permeation. See text and Table S1 for details.

In Figure 7, the result of the above analysis is graphically summarized. The most efficient inhibitors endowed with the highest CNS tropism are represented by points in the colored parallelepiped. The highest-ranking compound was BIA10-2474 (**21**). Thus, compounds ASP8477 (**10**), V158866 (**11**), and SA-57 (**20**), while displaying high efficiency and good CNS penetration, should be considered carefully for possible toxicity issues stemming from their profiles that seem close to the one of BIA10-2474 (**21**). Interestingly, compounds JNJ-42165279 (**7**) and MJN110 (**15**) are located near the high efficiency/selectivity/CNS permeation volume and may be considered a good compromise between efficiency and safety requirements. It is worth noting that CBD (**2**) is the less efficient inhibitor, highly promiscuous, and presents acceptable CNS permeation properties. This outcome agrees with what has been reported so far on CBD pharmacology.

## 6. Discussion

A relatively lower incidence of psychosis is found in Spain and Italy than in Northern Europe [134]. This evidence parallels the higher risk of developing schizophrenia among those born and brought up in cities than in rural settings [135]. The question is open if the green color itself may condition mental health [136]. It is perceived to be associated with life itself, and green color perception may boost the placebo effect when administering drugs acting on the CNS, such as tranquilizing medicines. It has been suggested that patients with schizophrenia should have more contact with green [137]. Ironically, it has been insinuated that a certain kind of ‘contact’ with ‘green’ would be mostly deleterious to our mind, with a clear winking to cannabis abuse in western cities as a triggering agent of psychosis [138]. Cannabis seems to have also deleterious long-term effects [139], thus dooming both adolescents and adults to the use of antipsychotics with related cardiometabolic liabilities due to adverse drug effects [140]. However, the ambiguity of cannabis makes it potentially both harmful to the heart [141] and beneficial in cardiovascular diseases [142]. The drug may have clinical applications for the treatment of autism spectrum disorder [143], and CBD showed antipsychotic potential. Since the intervention on ECS degrading enzymes is generally considered more suitable for ECS physiological balance restoration than the use of agents directly acting on CBR, we have tried to infer the possibility of repositioning some known endocannabinoid degradation enzyme inhibitors as potential antipsychotics, as considered by Navarrete et al. [144].

**Figure 8:**
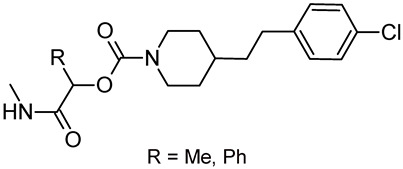
Possible chiral analogs of SA-57 (**20**).

Based on the performed predictions focused on CNS activity, oral adsorption, and ligand efficiency, the FAAH inhibitor JNJ-42165279 (**7**, Figure 3) seems to be the best candidate for this purpose, followed by the MGL inhibitor MJN110 (**15**, Figure 4). Other interesting inhibitors [e.g., ASP8477 (**10**), V158866 (**11**), ABX-1431 (**17**), and SA-57 (**20**)] may be considered as starting points for structural simplification/optimization endeavors to reduce possible hERG liability (**17**) and selectively favoring FAAH/MGL over other targets. None of the stated compounds is chiral, while chirality has been related to clinical success [145] and toxicity reduction [146]. Chiral analogs of the stated compounds could be easily designed and prepared to take advantage of chirality in the quest for safe and efficient inhibitors of FAAH and/or MGL as orally effective antipsychotics. Given the predicted favorable metabolic profile of SA-57 (**20**), lactamide (R = Me, Figure 8) and mandelamide (R = Ph, Figure 8) pyridyl carbamates could be considered as suitable starting points.

## Figures and Tables

**Table 1:** Results returned by the ligand-based classifier recently published by Delre et al. [120]. Notice that an IC_50_ = 10 μM was used as the threshold.

Target Enzyme	Inhibitor	Predicted hERG Blocking Ability
FAAH	URB597 (**5**)	Non-blocker
	URB937 (**6**)	Non-blocker
	JNJ-42165279 (**7**)	Non-blocker
	PF-04457845 (**8**)	Non-blocker
	SSR411298 (**9**)	Non-blocker
	ASP8477 (**10**)	Non-blocker
	V158866 (**11**)	Non-blocker
MGL	JZL184 (**12**)	Blocker
	KML29 (**13**)	Non-blocker
	URB602 (**14**)	Non-blocker
	MJN110 (**15**)	Non-blocker
	SAR127303 (**16**)	Non-blocker
	ABX-1431 (**17**)	Blocker
	PF-06795071(**18**)	Non-blocker
FAAH/MGL	JZL195 (**19**)	Blocker
	SA-57 (**20**)	Non-blocker

**Table 2:** Drug-likeness and ADME properties were computed for the compounds under investigation. Notice that the QED values were obtained by using an in-house script based on the paper by Bickerton et al. [126], while the software program QikProp, available from the Schrodinger suite 2022-4 [127], was employed to compute all the other relevant ADME properties. QED values > 0.6 are highlighted in bold.

	Compound	QED	CNS	QPP Caco *	QPlogBB **	Human Oral Absorption	Human Percent Oral Absorption	Rule of Three
FAAH Inhibitors	URB597 (**5**)	**0.89**	−2	142.196	−1.755	3	79.101	0
	URB937 (**6**)	**0.78**	−2	331.772	−1.286	3	89.452	0
	JNJ-42165279 (**7**)	**0.84**	2	373.210	0.496	3	87.382	0
	PF-04457845 (**8**)	0.57	0	509.513	−0.661	1	100.000	1
	SSR-411298 (**9**)	**0.66**	−2	65.327	−1.995	3	68.495	0
	ASP8477 (**10**)	**0.94**	0	1139.010	−0.644	3	100.000	0
	V158866 (**11**)	**0.78**	−1	964.142	−0.878	3	100.000	0
MGL Inhibitors	JZL184 (**12**)	0.39	−2	374.020	−1.335	2	72.038	1
	KML29 (**13**)	0.55	1	3100.818	0.221	1	100.000	1
	URB602 (**14**)	**0.84**	0	3551.379	−0.089	3	100.000	1
	MJN110 (**15**)	**0.65**	1	130.559	−0.400	3	85.779	0
	SAR127303 (**16**)	**0.64**	0	549.445	−0.372	3	100.000	0
	ABX-1431 (**17**)	0.53	2	1125.247	1.180	1	89.621	1
	PF-06795071 (**18**)	**0.80**	0	1152.477	−0.297	1	100.000	1
FAAH/ MGL Inhibitors	JZL195 (**19**)	0.41	0	131.320	−0.842	3	88.922	0
	SA-57 (**20**)	**0.90**	−1	538.896	−0.741	3	96.006	0
	BIA10-2474 (**21**)	**0.63**	0	854.649	−0.429	3	94.821	0
	CBD (**2**)	0.51	0	2405.636	−0.496	3	100.000	2

* QPPCaco: Predicted apparent Caco-2 cell permeability in nm/sec. ** QPlogBB: Logarithm of BBB predicted partition coefficient.

**Table 3:** Metabolic liability is predicted for all the investigated compounds. The prediction is performed using as software program CypReact.

	Compound	P450 Isoforms Predicted to React with the Ligand
FAAH Inhibitors	URB597 (**5**)	1A2 2C8 2C9 2C19 2D6 3A4
	URB937 (**6**)	1A2 2B6 2C8 2C9 2C19 2D6 3A4
	JNJ-42165279 (**7**)	01A2 2C8 2C9 2C19 2D6 3A4
	PF-04457845 (**8**)	1A2 2D6 3A4
	SSR-411298 (**9**)	1A2 2B6 2C8 2C9 2C19 2D6 3A4
	ASP8477 (**10**)	1A2 2B6 2C8 2C9 2C19 2D6 3A4
	V158866 (**11**)	2C8 2D6 3A4
MGL Inhibitors	JZL184 (**12**)	1A2 2C8 2C19 2D6 3A4
	KML29 (**13**)	1A2 2B6 2C9 2C19 2D6 3A4
	URB602 (**14**)	1A2 2B6 2C8 2C9 2C19 2D6 3A4
	MJN110 (**15**)	1A2 2B6 2C8 2C9 2C19 2D6 3A4
	SAR127303 (**16**)	1A2 2C8 2C9 2C19 2D6 3A4
	ABX-1431 (**17**)	1A2 2C8 2C9 2C19 2D6 3A4
	PF-06795071 (**18**)	1A2 2B6 2C8 2C9 2C19 2D6 2|E1 3A4
FAAH/ MGL Inhibitors	JZL195 (**19**)	None
	SA-57 (**20**)	None
	BIA10-2474 (**21**)	1A2 2C8 2C9 2C19 2D6 3A4
	CBD (**2**)	1A2 2B6 2C9 2C19 2D6 3A4

## Data Availability

Data sharing is not applicable.

## Supplementary Materials

The following supporting information can be downloaded at: https://www.mdpi.com/article/10.3390/biomedicines11020469/s1, Table S1: SMILES, Inhibiting Potency Values, Literature Sources of Data, and Ligand Efficiency Metrics for Compounds **2–21**.
